# Surgical repair for left coronary artery-right atrium fistula with giant coronary aneurysm

**DOI:** 10.1186/s13019-022-01857-z

**Published:** 2022-05-07

**Authors:** Yan Jin, Mengfei Zhang, Juan He

**Affiliations:** Department of Cardiology, West China Hospital, Sichuan University, Guoxuexiang 37th, Chengdu, 610041 Sichuan People’s Republic of China

**Keywords:** Coronary artery fistula, Coronary aneurysm, Surgical repair

## Abstract

**Background:**

Coronary artery fistula is a rare coronary anomaly which is defined as a communication between coronary artery and other heart chambers or vascular structures. The coronary artery which supply the fistula with blood can dilated, as a consequence, coronary aneurysm developed.

**Case introduction:**

Coronary artery fistula is frequently asymptomatic in its early stage, here we report a 26-year-old woman with left coronary artery fistula and left coronary artery aneurysm who presented in our hospital with dyspnea, fatigue and palpitation. The orifice of fistula was closed by continuous suture via right atriotomy. The wall of the aneurysm and enlarged LCA were partially resected along its longitudinal axis so that we can reduce the diameter of LCA to approximately normal.

**Conclusion:**

This technique provides a safe method for surgical repair of the giant coronary artery aneurysm with CAF.

## Background

Coronary artery fistula (CAF) is an uncommon heart anomaly which is defined as an abnormal connection between coronary artery and other heart chambers or vessels. The estimated prevalence of CAF is about 0.002% in general population and account for 0.2–0.4% of congenital heart disease and represent 14% of all the abnormalities of coronary [1, 2]. Ectasia of the coronary artery which supply the fistula with blood is a chronic effect which will lead to formation of coronary artery aneurysm [3]. CAF with coronary aneurysm is more prone to cause severe complications such as myocardial ischemia and aneurysm rupture so that it necessitates surgery treatment.

## Case report

**Fig. 1:**
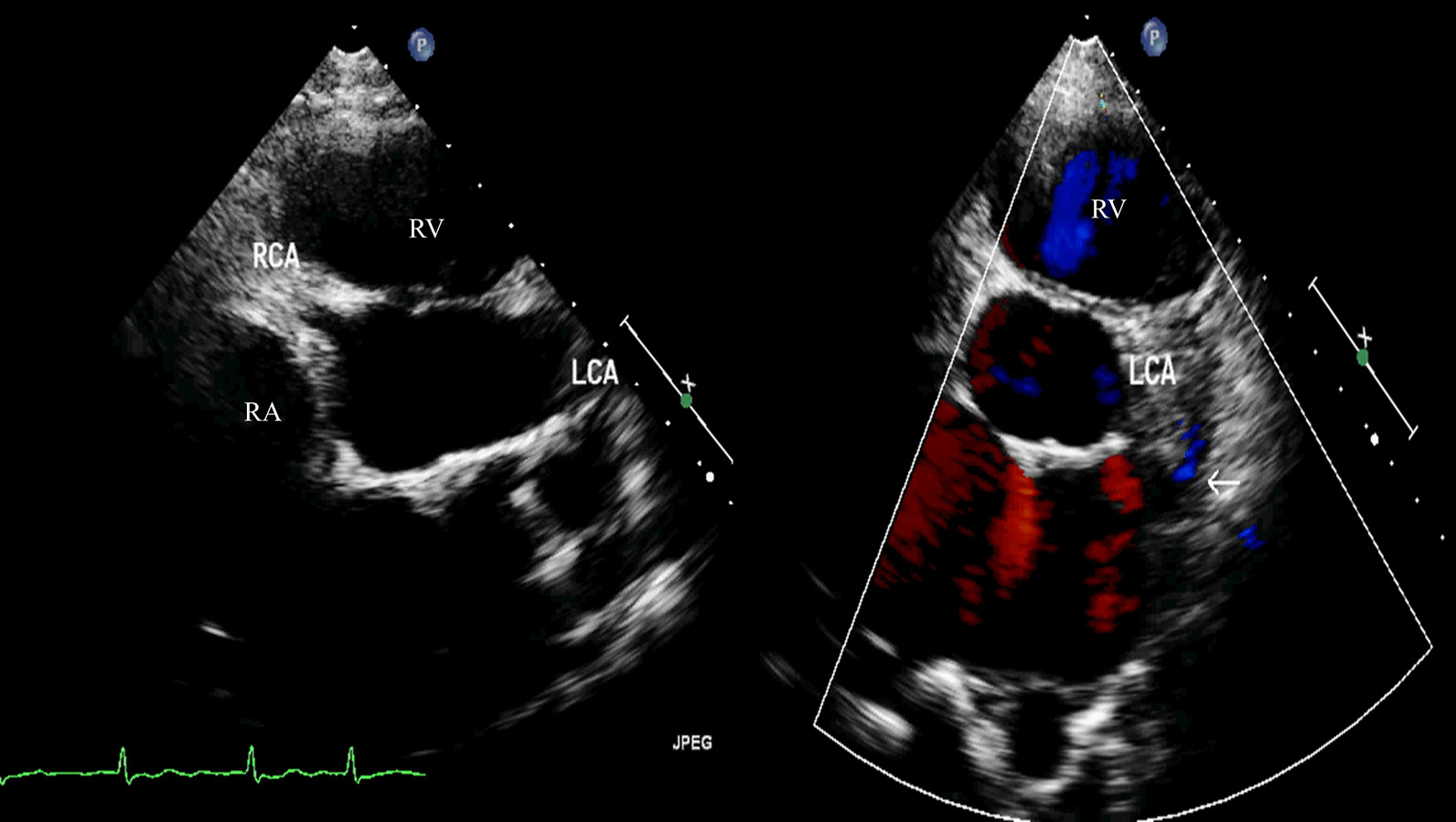
Transthoracic echocardiography showed a giant coronary aneurysm originated from distal left coronary artery and normal right coronary artery (LCA: Left coronary artery; RCA: Right coronary artery; RV: Right ventricle; RA: Right atrium)

**Fig. 2:**
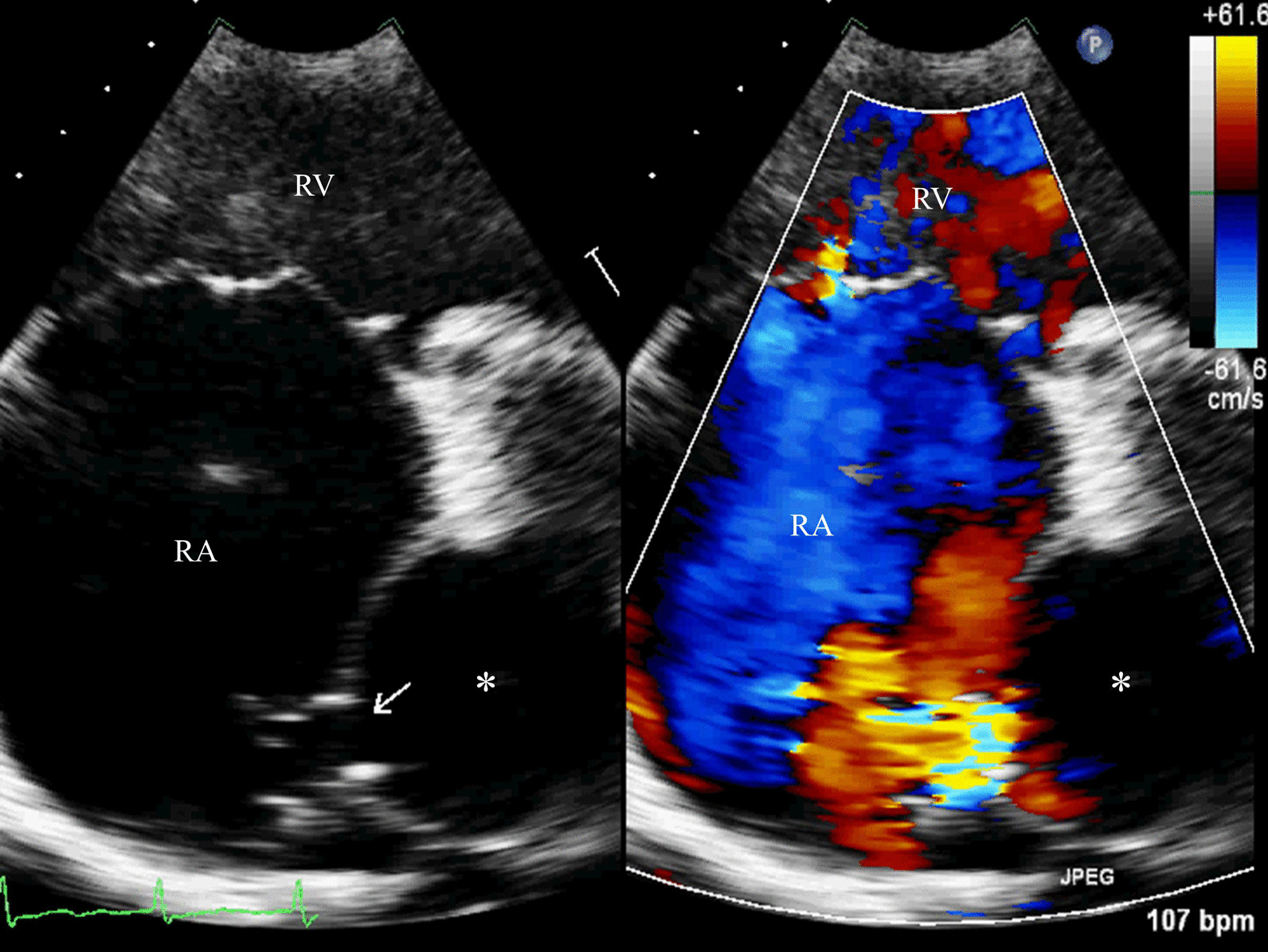
Transthoracic echocardiography revealed the LCA terminated in right atrium (arrow), proximal part of LCA was enlarged and the aneurysm (asterisk) was measured 56 × 45 mm (RV: Right ventricle; RA: Right atrium)

**Fig. 3:**
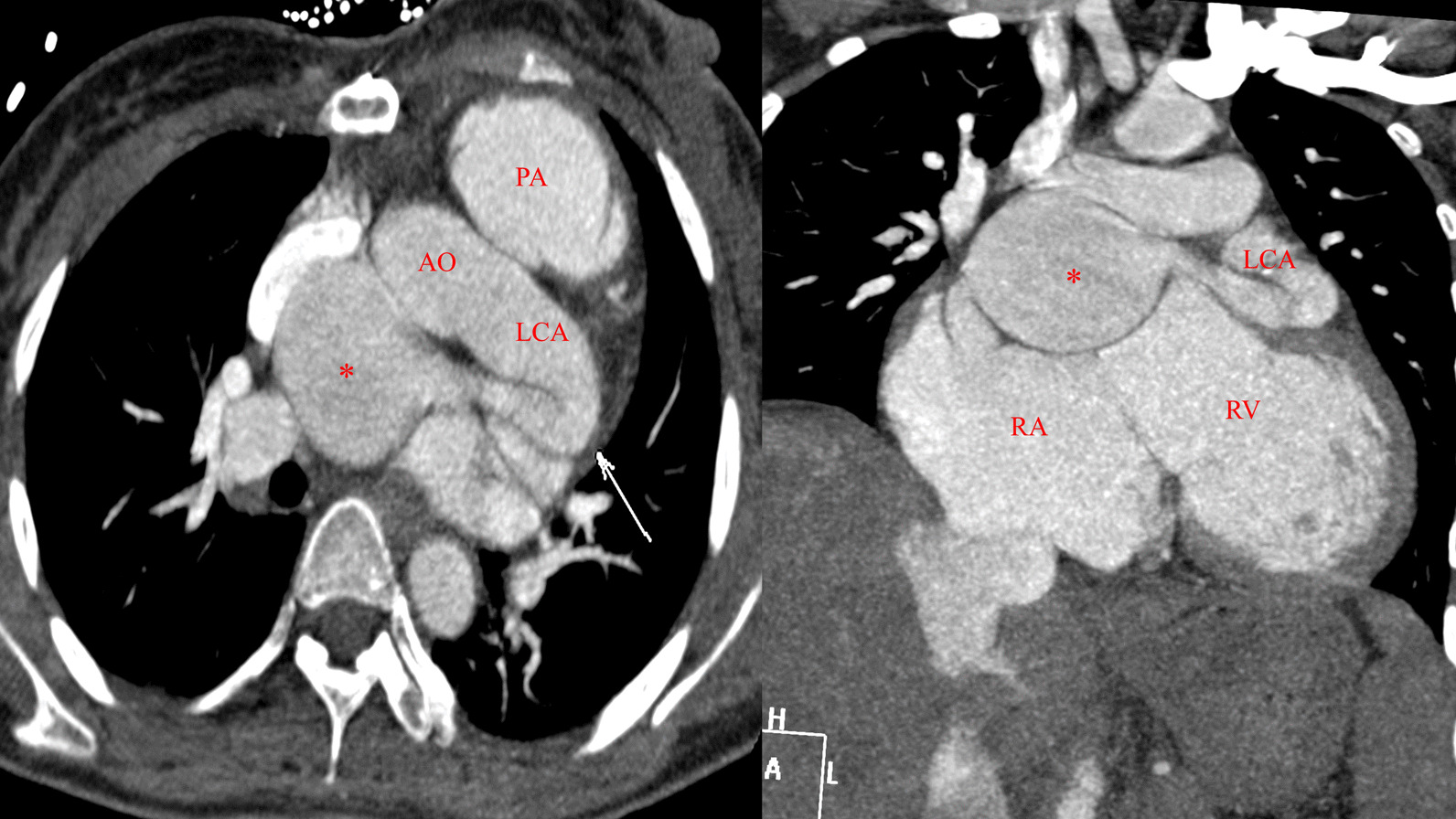
Multi-slice computed tomography angiography confirmed the diagnosis of LCA and revealed the significant enlargement of the LCA (arrow), the measured diameter of the coronary aneurysm (asterisk) was 45 × 56 mm and of the orifice of fistula was 9 mm (LCA: Left coronary artery; RV: Right ventricle; RA: Right atrium; AO: Aorta; PA: Pulmonary Artery)

A 26-year-old woman was referred to our hospital with dyspnea, fatigue and palpitation after exercise for a year. We found a continuous heart murmur during physical examination, heart beat was regular. Colored-Doppler echocardiography detected a giant coronary aneurysm originated from distal left coronary artery (LCA) (Fig. 1) and terminated in right atrium, proximal part of LCA was enlarged (Fig. 2). Multi-slice computed tomography angiography (CTA) and coronary angiography confirmed the diagnosis of CTA and revealed significant enlargement of LCA, the measured size of the coronary aneurysm was 45 × 56 mm and the diameter of the orifice of fistula was 9 mm (Fig. 3).

The surgery was performed through median sternotomy, cardiopulmonary bypass was initiated through aortic and bicaval cannulation. The temperature was decreased to 32 °C, and antegrade cardioplegia was administrated for cardiac arrest. The orifice of fistula was closed by continuous suture via right atriotomy. The wall of the aneurysm and enlarged LCA were partially resected along its longitudinal axis so that we can reduce the diameter of LCA to approximately normal. The condition of the patient in postoperative period was stable and postoperative echocardiography revealed no residual shunt of the fistula and each valve, she was discharged from the hospital uneventfully on the fifth postoperative day. The patient would be followed-up using CTA or echocardiography every three months in the first year after the operation.

## Discussion

The exact incidence of CAF is as yet unknown because the undiagnosed rate still remains high. Most of the CAF is asymptomatic and diagnosed incidentally during the routine physical examination. For the CAF can bring about bad consequences at an older age, the early diagnosis and treatment are very important.

CAF is usually classified into congenital CAF and acquired CAF, while congenital forms are more common, which is usually caused by the abnormal formation of the trabecular capillary network at the epicardial surface [4]. Acquired CAFs have been reported as a consequence of infective endocarditis, acute myocardial infarction, aortic dissection, and usually, iatrogenic causes [5]. Cardiac catheterization is considered to be the gold standard for the diagnosis of CAF, it can not only detect the cardiac structural involvement, but also achieve an interventional closure with a proper device [6]. Dual-source computed tomography (DSCT) has been widely used in clinical diagnosis of CAF because of its superiorities of short examining time, low radiation dose and excellent image quality [7]. Except that DSCT cannot be used to treat CAF, DSCT can prove detailed structural information about the fistula and concomitant cardiac malformations which are helpful for the therapeutic decision making. Echocardiography is the most common imaging modality for CAF owning to its repeatable and noninvasive features.

The treatment for the CAF is depended on characteristics of the fistula and the coronary artery, for most small CAFs and some medium-size asymptomatic CAFs, conservative treatment, including antiplatelet agents and regular follow-up. For most large CAFs and symptomatic CAFs, surgery is optimal treatment, which includes two main options: surgical procedure or transcatheter technique [8]. Simple CAF which means there is no coronary artery ectasia along the coronary that supply the fistula could be treated by transcatheter closure. The surgical intervention through which we can close the fistula, meanwhile fix the dilated coronary artery to approximately its normal size. In this case we chose surgical repair due to the giant coronary artery aneurysm, the risk of aneurysm rupture and thrombosis could be reduced to the most extent. Several surgical techniques have been reported and proved to be effective, besides ligation, patch repair and bypass graft, some novel techniques such as internal closure of the fistula and tangential arterial suture closure, have been described. Symbas et al. [9] reported a creation of a proximal arteriotomy and subsequent oversewing of the CAF origin intraluminally. May et al. reported a novel revascularization of distal posterolateral branches and bypass of internal thoracic artery graft [10].

## Conclusion

This technique provides a safe method for surgical repair of the giant coronary artery aneurysm with CAF.

## Data Availability

The datasets used are available from the corresponding author on reasonable request.

## Abbreviations

CAF: Coronary artery fistula
LCA: Left coronary artery
RCA: Right coronary artery
DSCT: Dual-source computed tomography
RV: Right ventricle
RA: Right atrium
AO: Aorta
PA: Pulmonary artery
